# Pleural mesothelioma incidence and use of systemic treatment decreased during the COVID-19 pandemic in The Netherlands

**DOI:** 10.1038/s41598-025-10054-6

**Published:** 2025-07-16

**Authors:** Illaa Smesseim, Li-Anne H. Douma, Jacobus A. Burgers, Ronald A. M. Damhuis

**Affiliations:** 1 Department of Thoracic Oncology, Netherlands Cancer Institute, Amsterdam, The Netherlands; 2 Department of Pulmonary Medicine, Leiden University Medical Center, Leiden, The Netherlands; 3 Department of Research and Development, Association of Comprehensive Cancer Centres, Utrecht, The Netherlands

**Keywords:** Mesothelioma, Covid-19, Pandemic, Asbestos, Pleural mesothelioma, Medical research, Oncology

## Abstract

Pleural mesothelioma (PM) is a lethal cancer often linked to asbestos exposure. The COVID-19 pandemic caused a global health crisis, raising concerns about its impact on cancer diagnoses and treatments. In response to the immense pressures on the healthcare system caused by the COVID-19 pandemic, many countries advised prioritizing essential healthcare services while postponing or suspending care considered non-emergent to prevent overburdening healthcare systems. This study assesses the impact of COVID-19 on the incidence, treatment, and overall survival of PM patients in the Netherlands between 2018 and 2022. Data were collected from the Netherlands Cancer Registry for 2,629 PM patients. Incidence, treatment patterns, and survival rates were analyzed using statistical methods, including Kruskal–Wallis and log-rank tests. PM incidence dropped 13.2% in 2020 during the pandemic, with a 58.8% increase in patients receiving best supportive care and a decline in chemotherapy use (from 39.4% to 32.0%). In 2021, diagnoses rebounded (+ 15.2%), and immunotherapy use rose following its approval. However, no significant difference in overall survival was found between 2018 and 2022. COVID-19 led to a temporary decline in PM diagnoses and systemic treatments in 2020, followed by recovery in 2021. Despite these changes, overall survival rates remained stable.

## Introduction

Pleural mesothelioma (PM) is a highly lethal tumor arising from serosal surfaces and often diagnosed at an advanced stage^1^. Asbestos exposure is linked to mesothelioma with a latency period of 20 to 50 years^2,3^. The overall clinical picture is characterized by atypical respiratory symptoms such as dyspnoea, cough and thoracic pain. This is known to lead to both a patient and a doctor’s delay^4^.

Global incidence rates vary between countries, with the highest rates in regions with a high development index, e.g. Northern Europe including the Netherlands, Australia and New-Zealand. In these regions in men the overall incidence started to decrease after the introduction of asbestos bans, whereas the incidence in female patients rose^5,6^.

More specifically, the incidence rates between 1993 and 2018 ranged between 2.6 and 4.1 cases per 100,000 person-years revised European standardized rate (RESR) in the Netherlands. Van Kooten et al.^7^ showed a significantly increased incidence at 1.6% annually (95% CI 1.0 to 2.1) up to 2010, followed by a non-significant decrease of − 1.7% annually (95% CI − 3.9 to 0.6).

Our research question is whether the incidence rate of mesothelioma over the past few years has been influenced by the global health crisis caused by the COVID-19 pandemic. In late 2019, several patients in China died from acute respiratory disease caused by severe acute respiratory syndrome coronavirus 2 (SARS-CoV-2). The virus rapidly spread across the globe, prompting the World Health Organization to officially declare the COVID-19 infection as a pandemic on March 11, 2020^8^. In response to the immense pressures on the healthcare system caused by the COVID-19 pandemic, many countries advised prioritizing essential healthcare services while postponing or suspending care considered non-emergent to prevent overburdening healthcare systems. Concurrently, some patients avoided hospital visits due to concerns about the risk of virus transmission. The American Medical Association provided guidance to healthcare workers on delaying and resuming elective procedures during the pandemic, which led to significant backlogs and delays in healthcare^9,10^. In August 2020, the World Health Organization reported that 89% of the 105 countries surveyed experienced disruptions to essential health services^11^. In the Netherlands, this was reflected in a noticeable decrease in cancer diagnoses in 2020^12^.

**Fig. 1:**
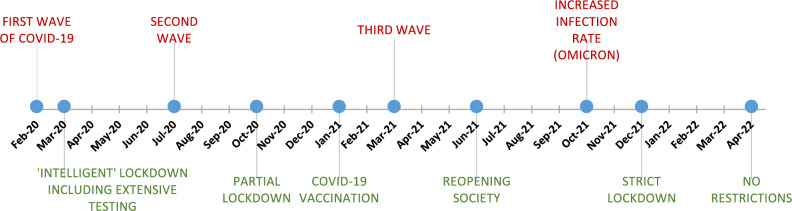
COVID-19 outbreaks and measures in the Netherlands.

The timeline of COVID-19 events in the Netherlands, from the first diagnosis in February 2020 through multiple waves, lockdowns, and vaccination efforts until the lifting of most restrictions by April 2022, is summarized in Fig. 1. On May 5, 2023, the WHO declared that COVID-19 was no longer a global health emergency^13,14^.

The Dutch Society of Pulmonology and Tuberculosis (NVALT) recommended in 2020 to postpone cytotoxic chemotherapy for as long as possible when considered acceptable from a medical perspective. This led to a temporary decline in new lung cancer diagnoses in 2020 compared with 2019^15^. Fazzo et al. investigated mortality rates from asbestos-related diseases in Italy, the first European country significantly affected by the COVID-19 pandemic, during the pandemic’s first year. The study focused on malignant mesothelioma (PM) mortality in 2020 and its relationship with COVID-19. By comparing mortality rates from 2020 to those recorded from 2015 to 2019, the researchers found a relative mortality risk increase of 1.10 among patients aged 80 years and older. Also, they identified a decrease in hospitalizations for PM across all age groups in 2020 compared to the period from 2010 to 2019^16^.

The aim of this study was to assess the impact of the COVID‐19 pandemic on the (1) incidence of PM in the Netherlands, (2) treatment and (3) overall survival.

## Methods

### Data collection

Patients older than 18 years diagnosed with pleural mesothelioma between 2018 and 2022 were selected for inclusion from the Netherlands Cancer Registry (NCR). Patients with mesothelioma in non-pleural sites were not included in this study. The NCR has records of all newly diagnosed pleural mesothelioma patients notified by the Dutch Pathological Anatomical National Automated Archive and the National Registry of Hospital Discharge Diagnoses. The NCR is hosted by the Netherlands Comprehensive Cancer Organization (IKNL). This study is retrospective in nature, and the requirement for informed consent was waived by the Ethics Review Committee of the Netherlands Cancer Registry, under approval number [K22.180_2]. The following data were retrieved: age at diagnosis, sex, detection method (cytology, histology of primary tumor or metastasis), tumour characteristics (e.g., TNM stage, histology), overall survival and primary treatment (systemic chemotherapy, immunotherapy, surgical treatment, radiotherapy or best supportive care). The overall survival was calculated from date of diagnosis to the date of death or the date of censoring (1^st^ February 2024). All methods were conducted in compliance with the declaration of Helsinki.

### Statistical analyses

Differences between multiple groups were tested for significance using the Kruskal–Wallis test for median age and time from diagnosis to treatment in days and one-way ANOVA for all other variables. Variation in survival rates was assessed by the log-rank test. For all tests, two-sided *P*-values of < 0.05 were considered statistically significant. All statistical analyses were performed using SPSS Statistics software (version 20.0; IBM, Armonk, NY) and RStudio 2023.03.0 + 386 “Cherry Blossom”.

## Results

**Fig. 2:**
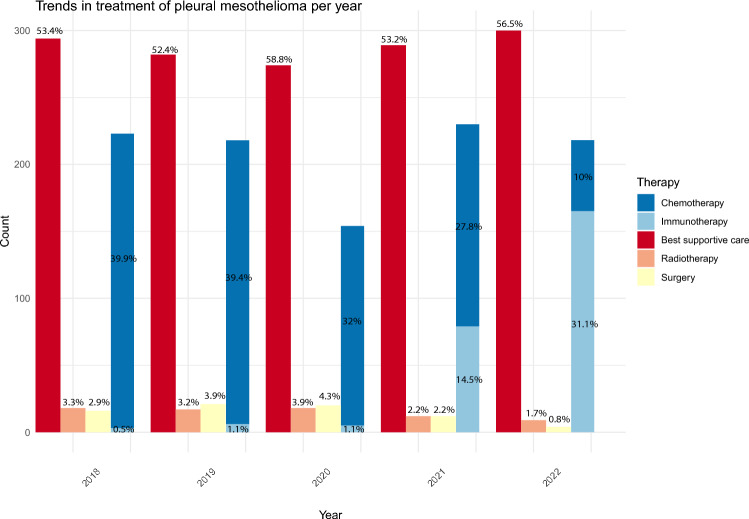
Trends in treatment of pleural mesothelioma.

There were 2629 patients with PM identified between 2018 and 2022. The PM incidence per 100,000 inhabitants was 3.21, 3.11, 2.70, 3.11, and 3.02 in 2018, 2019, 2020, 2021, and 2022, respectively. A comprehensive overview of the patient and tumor characteristics at the time of diagnosis is provided in Table 1. The group was predominantly male, with percentages ranging from 82.3% to 87.5%, and the median age ranged from 75 to 77 years. The ratio between male and female patients remained stable throughout the years. The number of newly diagnosed mesothelioma patients decreased by 3.12% from 2018 to 2019. The following year, the decline was higher at 13.2%. Comparing 2020 with 2021, there was a significant increase in the number of new diagnoses per 100,000 people of 15.2%, followed by a decrease of 2.9% the subsequent year. An overview of the trends in treatment is provided in Fig. 2.

**Table 1:** Patient and tumour characteristics at the time of diagnosis.

	2018 (n = 551)	2019 (n = 538)	2020 (n = 466)	2021 (n = 543)	2022 (n = 531)	p-value
Age (median, IQR)	75 (69–81)	75 (70–80)	76 (71–80)	76 (72–81)	77 (72–82)	< 0.001
Sex (n,%)						
Male	482 (87.5%)	443 (82.3%)	398 (85.4%)	464 (85.5%)	460 (86.6%)	0.159
Female	69 (12.5%)	95 (17.7%)	68 (14.6%)	79 (14.5%)	71 (13.4%)	
Detection method						
Cytology	98 (17.8%)	92 (17.1%)	75 (16.1%)	113 (20.8%)	107 (20.2%)	0.275
Histology of metastasis	14 (2.5%)	14 (2.6%)	12 (2.6%)	12 (2.2%)	13 (2.4%)	
Histology of primary tumour	439 (79.7%)	432 (80.3%)	379 (81.3%)	418 (77.0%)	411 (77.4%)	
Primary treatment						
Chemotherapy	220 (39.9%)	212 (39.4%)	149 (32.0%)	151 (27.8%)	53 (10.0%)	< 0.001
Radiotherapy	18 (3.3%)	17 (3.2%)	18 (3.9%)	12 (2.2%)	9 (1.7%)	
Surgery	16 (2.9%)	21 (3.9%)	20 (4.3%)	12 (2.2%)	4 (0.8%)	
Immunotherapy	3 (0.5%)	6 (1.1%)	5 (1.1%)	79 (14.5%)	165 (31.1%)	
Best supportive care	294 (53.4%)	282 (52.4%)	274 (58.8%)	289 (53.2%)	300 (56.5%)	
Histology						
Nonspecific	102 (18.5%)	108 (44.1%)	76 (14.0%)	76 (14.0%)	81 (15.3%)	0.260
Sarcomatoid	90 (16.3%)	96 (17.8%)	91 (19.5%)	94 (17.3%)	90 (16.9%)	
Epithelioid	322 (58.4%%)	283 (52.6%)	263 (56.4%)	334 (61.5%)	318 (59.9%)	
Biphasic mesothelioma	37 (6.7%)	51 (9.5%)	45 (9.7%)	39 (7.2%)	42 (7.9%)

The majority of the patients with PM received best supportive care which peaked in 2020 (58.8%). Also, less patients were treated with chemotherapy in 2020 in comparison to the previous year (decreasing from 39.4 to 32.0%). Although in 2021 a similar number of patients was treated with chemotherapy as in 2020, more patients received immunotherapy (14.5%) after its use was formally approved in the Netherlands and in a compassionate use program administered in two expert centers in the Netherlands (Fig. 2).

The percentage of total PM patients (n = 2629) who had died in the consecutive years (2018–2022) was 20.7%, 21.3%, 17.6%, 19.8%, and 20.6% (*p* = 0.19). The median overall survival for the years 2018–2022 was as follows: 9.0 months (IQR: 4.0–18.0), 9.0 months (IQR: 4.0–18.3), 8.5 months (IQR: 3.0–20.0), 10.0 months (IQR: 3.0–16.0), and 9.0 months (IQR: 3.0–16.0).

**Fig. 3:**
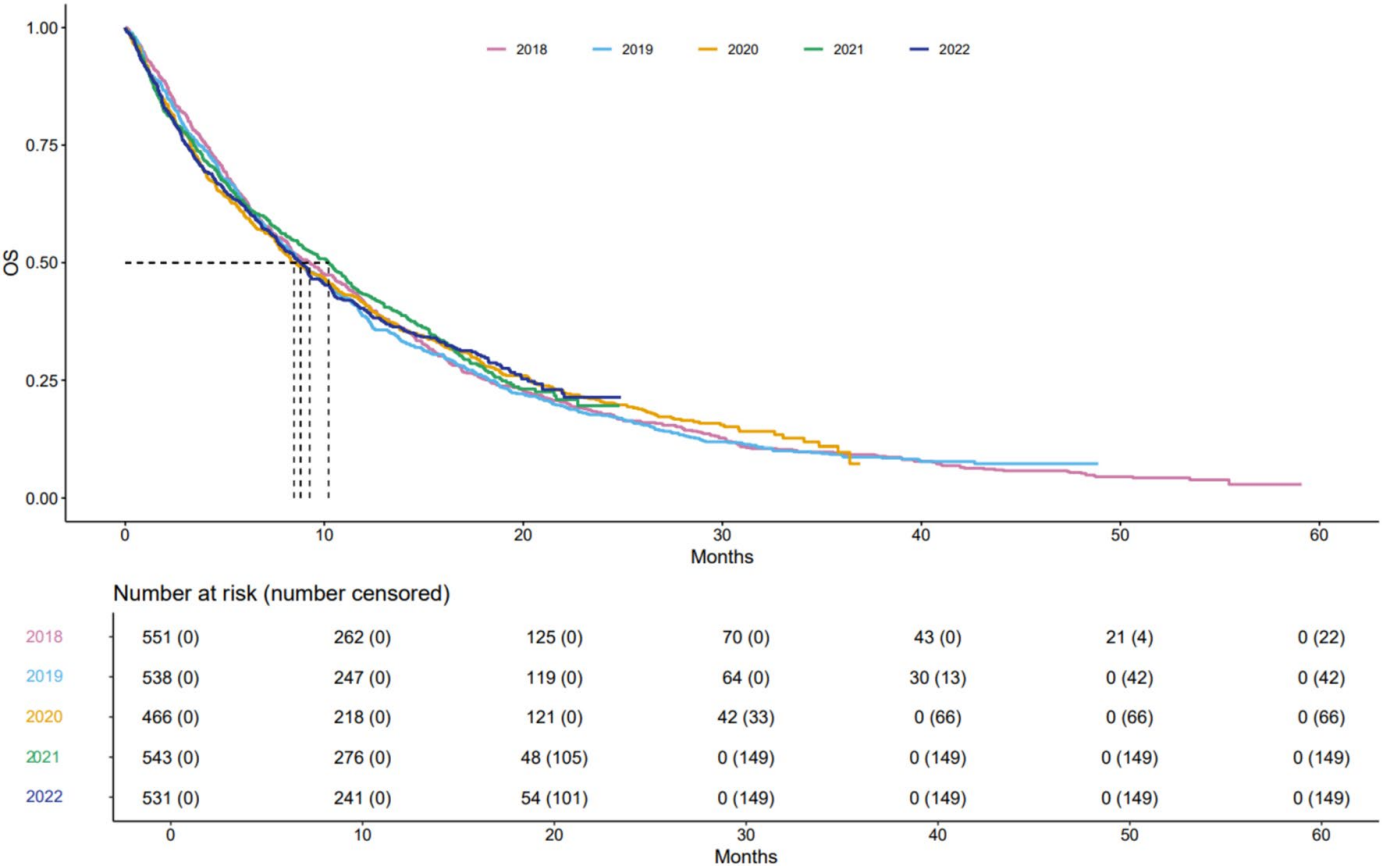
Kaplan–Meier curves of overall survival of all patients per year Stratified by year of diagnosis. Log-rank test: Chisq = 0.9 on 4 degrees of freedom, *p* = 0.90.

**Fig. 4:**
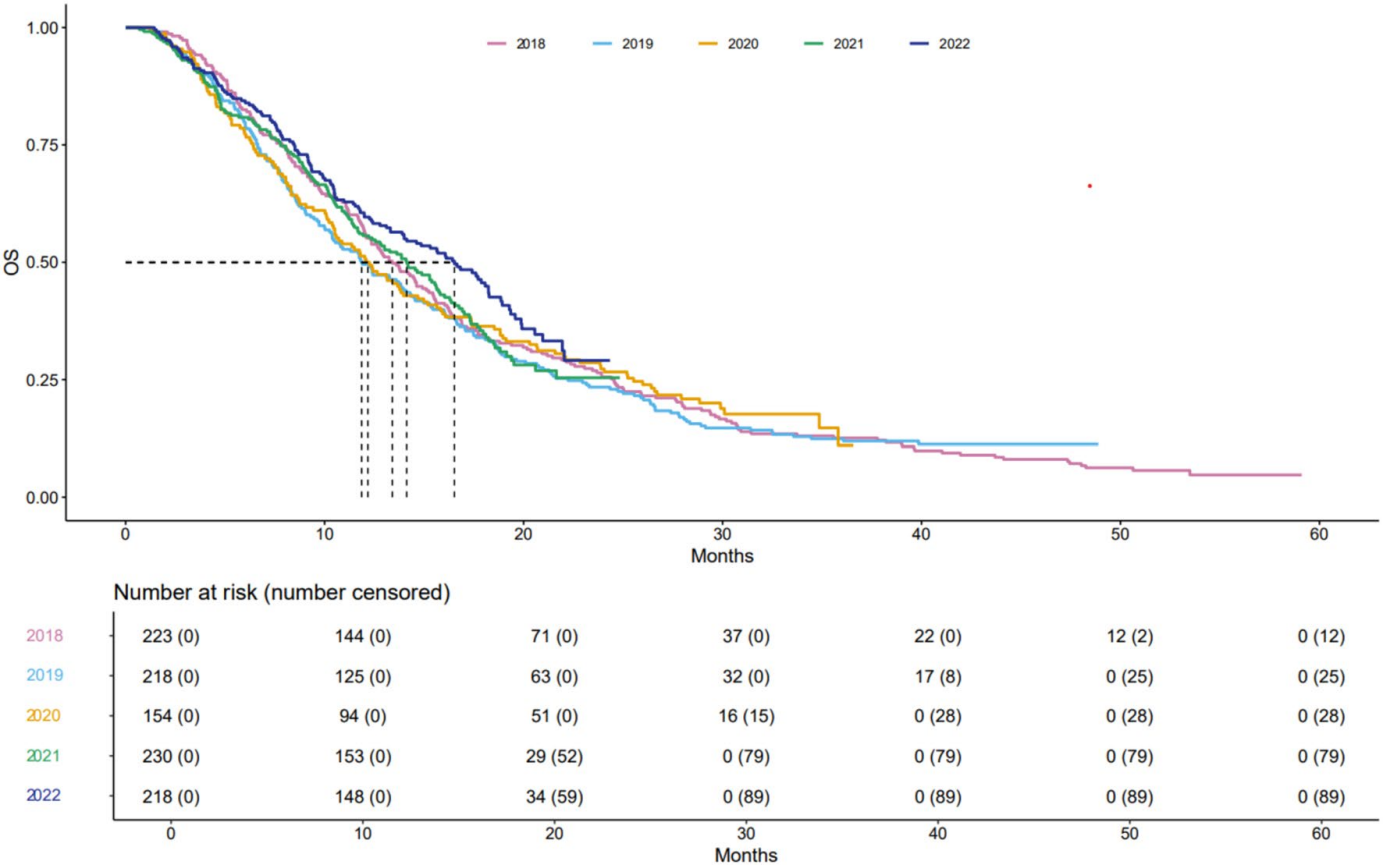
Kaplan–Meier curves of overall survival of patients with pleural mesothelioma who received systemic treatment per year. Log-rank test: Chisq = 4.9 on 4 degrees of freedom, *p* = 0.30.

There was no statistically significant difference in overall survival of patients diagnosed with PM between 2018 and 2022 (*p* = 0.90); see Table 1 and Fig. 3. Similarly, there was no statistically significant difference in overall survival among patients diagnosed with PM between 2018 and 2022 who were treated with systemic therapy (*p *= 0.30); see Fig. 4. Despite the lack of significant results we did observe a mild upward trend in 2021 and 2022.

## Discussion

In 2020, during the COVID-19 pandemic, there was a 13.2% decline in the number of newly diagnosed mesothelioma cases per 100,000 people. The highest percentage of patients treated with best supportive care was recorded that year at 58.8%. This did not result in a significant difference in overall survival between 2018 and 2022. From 2020 to 2021, the number of new diagnoses per 100,000 bounced back by 15.2%. In that year, the number of patients receiving immunotherapy began to rise following its approval in the Netherlands, while the number of patients treated with chemotherapy started to decline. Before 2018, Kooten et al. reported that the incidence of PM increased steadily by approximately 1.8% per year (95% CI 1.1–2.5) between 1989 and 2007, with rates ranging from 2.4 to 3.5 cases per 100,000 person-years (RESR). After 2007, they noted a non-significant decline in incidence, with incidence rates between 3.3 and 3.8 cases per 100,000 person-years (RESR). Compared to these earlier data, the annual incidence of PM from 2018 to 2022 appears lower than in previous years.

During the pandemic, patients were advised to consult the general practitioners only for urgent cases, potentially resulting in delays in cancer diagnosis. Furthermore, a doctor’s delay might have occurred, because patients’ symptoms could have been mistaken for COVID-19, resulting in postponements of referrals to pulmonologists. Additionally, there was a tendency not to conduct pathology diagnostics during the acute phase of COVID-19. Presumably, some mesothelioma patients were not recorded in the Netherlands Cancer Registry but were instead documented under a COVID diagnosis in mortality statistics.

Moreover, in 2020, less patients received chemotherapy (32.0 vs 39.4%) compared to the preceding year, in alignment with the advice of the NVALT recommendations to postpone chemotherapy for mildly symptomatic patients. The number of patients treated with systemic treatment returned to normal in 2021. During that year, immunotherapy was introduced as the first-line treatment for PM patients.

However, despite this novel treatment regimen, no significant survival benefit was shown. This could be attributed to differences between real-world survival data and those observed in clinical studies. The CheckMate 743 trial showed that patients with PM treated with first-line ipilimumab and nivolumab had a median overall survival of 18.1 months (95% CI 16.8–21.0 months). However, real-world cohorts have shown lower survival outcomes. For example, Schmid et al. reported a median overall survival of 12.6 months (95% CI 6.5–16.5) in Switzerland, McNamee et al. reported 14.5 months (95% CI 12.5–NR) in Australia, and the FLORA study found a median overall survival of 14.6 months (95% CI 12.7–16.5) in The Netherlands.

Another observation in our dataset shows that there is a trend towards less surgical procedures in 2022, possibly due to the pandemic. Importantly, in the Netherlands, systemic treatment forms the backbone of therapy, with surgery playing a very limited role.

This study had several limitations. One key limitation is the potential lack of generalizability, as mesothelioma care is not uniformly regulated across countries. In the Netherlands, care for pleural mesothelioma (PM) patients is centralized in two recognized expert centers. However, the organization of PM patient care may differ significantly from other countries. Another limitation of this study is the effect of underdiagnosis of PM due to a diagnostic gap caused by limited access to diagnostic procedures during the pandemic and patients’ avoidance of hospitals out of fear of infection. Since respiratory symptoms of PM can overlap with those of COVID-19, it is possible that some PM symptoms were mistakenly attributed to COVID-19.

It is beyond the scope of this study to determine whether the decline in systemic therapy has led to a poorer survival rate. A strength of the study is its limited selection bias, facilitated by data acquisition from a national registry (IKNL) that retrieves information regarding all patients.

## Conclusion

This nationwide observational cohort study demonstrates that, following the onset of the COVID-19 pandemic, there was an abrupt decline in the incidence and systemic treatment of PM patients in 2020. This was followed by an increase in PM incidence, the number of patients treated with systemic therapy, and overall survival in the subsequent year. No significant difference in overall survival was observed.

## Data Availability

The datasets generated and/or analysed during the current study are not publicly available due to privacy restrictions but are available from the corresponding author on reasonable request.
